# Clinical and radiological results of allogenous bone graft versus synthetic calcium‐phosphate graft in opening wedge high tibial osteotomy

**DOI:** 10.1002/jeo2.70592

**Published:** 2026-02-10

**Authors:** Yoosef Mehrabi, Salar Baghbani, Seyyed Reza Sharifzadeh, Davood Feizi, Saeed Hesaraki, Saeed Bakhshi, Mohammad Movahedinia, Mostafa Shahrezaee

**Affiliations:** ^1^ Department of Orthopedic Surgery, Shariati Hospital Tehran University of Medical Sciences Tehran Iran; ^2^ Orthopedic Surgery Ward, Besat Hospital, Trauma and Surgery Research Center AJA University of Medical Sciences Tehran Iran; ^3^ Department of Orthopedic Surgery AJA University of Medical Sciences Tehran Iran; ^4^ Biomaterials Group, Nanotechnology and Advanced Materials Department Materials & Energy Research Center Alborz Iran; ^5^ Cancer Research Center Tehran University of Medical Sciences Tehran Iran; ^6^ Department of Orthopedic Surgery Shahid Beheshti University of Medical Sciences Tehran Iran

**Keywords:** allogenic graft, calcium‐phosphate synthetic granules, opening wedge high tibial osteotomy, varus deformity

## Abstract

**Purpose:**

To compare the clinical and radiographic data after opening wedge high tibial osteotomy (OWHTO) using an allogenous bone graft versus a tricalcium phosphate (TCP) graft.

**Methods:**

This prospective clinical study enroled 42 patients (84 knees) between September 2022 and March 2023, undergoing bilateral OWHTO for genu varum. Patients' knees were randomized intraoperatively into two groups: those receiving an allograft or TCP graft to fill the osteotomy defect. Preoperative assessments included pain severity via the visual analogue scale (VAS) and joint function using the Western Ontario and McMaster University Osteoarthritis Index (WOMAC). The osteotomy was corrected using the Minami–Miniaci method, and stabilization was achieved with a fixed‐angle locking plate. Pain, complications and radiographic outcomes were monitored post‐operatively, with bone union assessed via the Hemert criteria.

**Results:**

In this cohort of 42 patients (59.5% male, mean age 36.6 ± 5.0 years), all non‐smokers, the mean follow‐up was 2.1 years. Pre‐ and post‐operative comparison using WOMAC (pre‐op = 16.1 ± 8.4; post‐op = allograft: 11.3 ± 4.7, *p* = 0.00; TCP = 9.3 ± 4.8, *p* = 0.00) and VAS scores (pre‐op = 2.8 ± 0.9; post‐op = allograft: 2.2 ± 0.8, *p* = 0.00; TCP = 1.8 ± 1.2, *p* = 0.00) revealed statistically and clinically significant outcomes. In WOMAC components meaningful pain (pre‐op = 4.4 ± 1.7; post‐op = allograft: 2.9 ± 1.3, *p* = 0.00; TCP = 2.4 ± 1.4, *p* = 0.00), stiffness (pre‐op = 1.7 ± 1.2; TCP = 1.3 ± 1.1, *p* = 0.01) and physical function (pre‐op = 9.9 ± 7.1; post‐op = allograft: 7.7 ± 3.9, *p* = 0.00; TCP = 6.4 ± 4.0, *p* = 0.00) improvements at 12 months in both grafts, except stiffness in allograft group (pre‐op = 1.7 ± 1.2 post‐op = allograft: 1.6 ± 1.2, *p* = 0.08). However, post‐operative analysis revealed that the TCP graft showed statistically significant superiority in WOMAC and VAS scores (*p* < 0.05), except pain component of WOMAC (TCP = 2.4 ± 1.4, allograft = 2.9 ± 1.3, *p* = 0.305). Bone consolidation occurred in 54.8% of cases. No intraoperative complications, infections or loss of correction were reported during follow‐up.

**Conclusions:**

Patients undergoing OWHTO with TCP granules experienced better outcomes and less pain, compared to those receiving allogeneic bone grafts.

**Level of Evidence:**

N/A.

## Graphical abstract

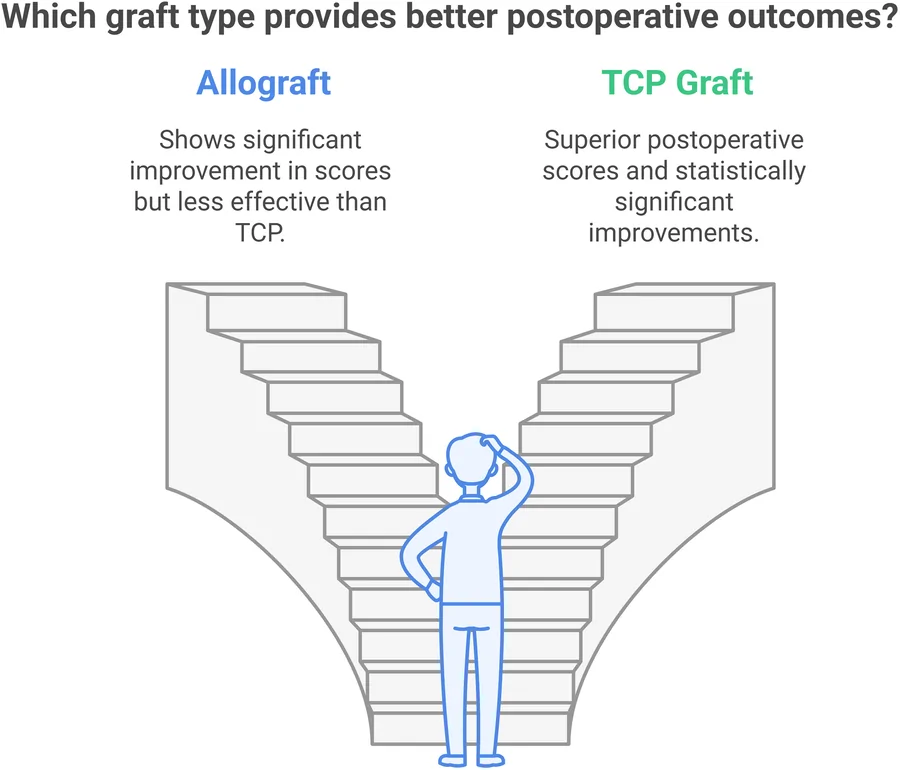

**Abbreviations**
BMI: body mass index
CT: computed tomography
HKA: hip–knee–ankle
KSFS: Knee Society Functional Score assessment
KSS: Knee Society Score
LDFA: lateral distal femoral angle
MPTA: medial proximal tibial angle
OWHTO: opening wedge high tibial osteotomy
ROM: range of motion
TCP: tricalcium phosphate
VAS: visual analogue scale
VD: varus deformity
WOMAC: Western Ontario and McMaster University Osteoarthritis Index

## INTRODUCTION

High tibial osteotomy is primarily indicated for physically active patients with medial compartment osteoarthritis associated with a varus deformity (VD) of tibial origin [37, 50]. The opening wedge high tibial osteotomy (OWHTO) technique has been widely used to correct angular deformity while preserving bone stock and reducing the risk of peroneal nerve injury, which may occur after fibular osteotomy [17, 51]. However, OWHTO creates an osseous gap that requires osteogenesis to resolve the defect. The debate continues over whether this gap should be filled with grafts or artificial substitutes [10]. While some authors suggest that minor osseous defects do not require grafting, many agree that significant defects should be augmented with autografts, allografts or artificial substitutes, especially in cases of severe VD, obesity and poor bone quality [18, 26, 29, 47].

Despite grafting, delayed osseous consolidation has been reported in up to 10% of cases and is associated with prosthetic failure and loss of angular correction, leading to poor clinical outcomes [12, 22]. Autografts promote osteoinduction and osteoconduction and are considered the gold standard among graft materials for treating defects in fractures or nonunions. However, significant disadvantages include donor site morbidity, reported in 0.76%–26% of patients, as well as increased post‐operative pain, haemorrhage and longer operative duration [5, 19, 23, 30, 42]. Consequently, allografts or artificial bone substitutes, such as hydroxyapatite or tricalcium phosphate (TCP), are predominantly used instead of autografts [1]. Studies have shown that allografts in OWHTO yield more acceptable clinical results than autografts, except in cases of lateral cortical breakage [6, 13, 16, 38, 39]. Although allografts have been used in OWHTO for an extended period, there are inherent risks related to immunogenic responses and pathogen transmission [2].

While several clinical reports and comparative studies have attempted to evaluate the effectiveness of bone graft materials in OWHTO [12, 19], a direct comparison between allogenous bone grafts and TCP substitutes is notably absent. This study aims to fill this gap in the literature. Most research has focused on clinical outcomes and loss of correction, but very few studies have assessed patient performance after OWHTO. Additionally, almost all comparative studies have utilized wedge‐type grafts or substitutes, with limited use of granule‐type graft materials [8].

The current study compares the clinical and radiographic outcomes after OWHTO using allogenous bone grafts versus TCP granules as grafting materials. One hypothesis is that the progression of osseous union in OWHTO treated with TCP granule grafting would not differ from that in OWHTO treated with allogenous bone grafting. The primary purpose of this study is to demonstrate that TCP granules could serve as a promising and potentially game‐changing bone substitute, mitigating some drawbacks associated with allogeneic bone grafts.

## PATIENTS AND METHODS

### Patient cohort

Between September 2022 and March 2023, 42 individuals (84 knees) undergoing bilateral OWHTO were prospectively enroled in this clinical investigation. The minimum duration of post‐operative follow‐up was 12 months (ranging from 12 to 54 months), with a complete retention rate for all subjects. Adult patients aged 22–45 years, presenting with genu varum and eligible for OWHTO, were included after informed consent was obtained in accordance with the Declaration of Helsinki. Institutional review board approval was secured from our affiliated tertiary care academic hospital.

Exclusion criteria included subjects with moderate to severe osteoarthritic manifestations in the medial compartment of the knee, based on Ahlbäck criteria; knee range of motion (ROM) of less than 100°; flexion contracture exceeding 15°; a history of rheumatoid arthritis or other systemic inflammatory arthropathies; lateral tibiofemoral subluxation greater than 1 cm; VD surpassing 15°; the presence of lateral thrust during gait; body mass index (BMI) exceeding 35; prior surgical intervention or infection in the knee joint; chondrocalcinosis; and chronic corticosteroid administration.

### Preoperative evaluation and surgical planning

Pain severity was quantitatively assessed using a visual analogue scale (VAS; 0 = *no pain*, 10 = *maximal conceivable pain*). Functional disability and joint‐specific symptoms were systematically evaluated with the Western Ontario and McMaster Universities Osteoarthritis Index (WOMAC). The attending orthopaedic surgeon conducted a comprehensive preoperative clinical and radiographic assessment. Imaging included standard anteroposterior and lateral knee radiographs to determine the medial proximal tibial angle (MPTA), lateral distal femoral angle (LDFA), hip–knee–ankle (HKA) angle, VD and posterior tibial slope. Knee ROM was accurately measured using a calibrated goniometer. The surgical objective was to restore the HKA angle to within 182–186° (corresponding to 2–6° of post‐operative valgus alignment). The correction magnitude was derived from full‐length weight‐bearing radiographs using the Minami–Miniaci method and transposed to osteotomy height via the Hernigou–Goutallier conversion tables.

In the preoperative phase, tranexamic acid and cefazolin were administered intravenously to reduce intraoperative blood loss and mitigate infection risks. Regional anaesthesia was achieved through spinal blockade, and a pneumatic tourniquet was applied to the proximal thigh to maintain a pressure of 270 mmHg throughout the procedure.

### Randomization and surgical technique

Randomization occurred intraoperatively after the completion of osteotomy, during which the patient's lower extremity side was allocated to either the allograft group or the TCP group. The osteotomy gap on one side was packed with an allogenous tricortical bone graft (Iranian Tissue Bank). On the TCP graft side, ceramic granules composed of 100% pure beta‐tricalcium phosphate (Ca_3_(PO_4_)_2_), sintered at 1200°C, were used to fill the osteotomy defect. These ceramic granules were available in standardized dimensions.

The osteotomy was performed using a biplanar technique via a medial proximal incision, with careful preservation of the lateral cortical hinge. The osteotomy was opened incrementally with a lamina spreader until the desired correction was achieved, as predetermined by preoperative planning. Mechanical axis realignment was confirmed intraoperatively with a radiopaque wire under fluoroscopic guidance (C‐ARM), ensuring that the corrected axis traversed the midline of the knee joint. Upon verification of satisfactory alignment, stabilization of osteotomy was achieved using a fixed‐angle locking plate, and the gap was filled with the allocated graft material. The surgical wound was sutured in layers and dressed in sterile bandages. A standardized fixation protocol involving a fixed‐angle locking plate was applied in all cases.

### Post‐operative management and follow‐up

Post‐operative management commenced within 24 h following surgery, with early mobilization facilitated by a hinged knee brace. Partial weight‐bearing ambulation was permitted. Post‐operative assessments were scheduled at 2 weeks to evaluate wound healing, with subsequent visits at 1, 3 and 6 months for radiological and clinical evaluations conducted by an independent examiner. Pain intensity was reassessed at each follow‐up using the VAS. Detailed documentation of adverse events, perioperative complications and surgical variables, including operative time and length of hospital stay, was maintained. Follow‐up imaging included anteroposterior and lateral knee radiographs at 1 and 6 months, HKA radiographs at 3 months, and a computed tomography (CT) scan at the final follow‐up. Loss of correction was defined as any post‐operative displacement of the osteotomy site prior to union or a reduction of ≥3° in the HKA angle on standing leg radiographs. Two independent orthopaedic surgeons assessed bone union and graft integration using the Hemert criteria, with union defined at stages 3 or 4.

### Statistical analysis

Quantitative variables were analyzed using the Student's *t* test, while qualitative outcomes were evaluated with the chi‐square test, applying Fisher's exact correction where appropriate. The effect of graft type (allograft vs. TCP graft) on post‐operative outcomes, including pain relief, union rates and complication incidence, was assessed. Statistical significance was defined as *p* values < 0.05. Power analysis determined a minimum cohort size of 42 patients and 84 knees to detect clinically significant differences in outcomes, with an 80% power (*β* = 0.2) and a type I error rate of 5%. Statistical analyses were performed using SPSS‐29 software for Windows.

## RESULTS

### Population

A cohort of 42 patients was enroled, with a mean follow‐up duration of 2.1 years (1–4.5 years). The cohort included 25 males (59.5%) and 17 females (40.5%), with a mean age of 36.6 ± 5.0 years (range: 22–45 years). All participants were non‐smokers. In 50% (21 cases) of the patients, the initial osteotomy was performed on the left side, followed sequentially by the surgical intervention on the contralateral right side (Table 1). Among the cohort of 84 knees subjected to OWHTO, autologous structural augmentation was achieved using allogeneic bone grafts in 42 cases (50%), while the remaining 42 knees (50%) were reconstructed utilizing TCP synthetic grafts as osteoconductive scaffolds.

**Table 1:** Patient's characteristics.

Variables	Statistics
Age (years; mean ± SD)	36.6 ± 5.0
Gender	
Male (*N*; %)	25 (59.5)
Female (*N*; %)	17 (40.5)
BMI (kg/m^2^; mean ± SD)	27.5 ± 2.8
First side	
Right (*N*; %)	21 (50.0)
Left (*N*; %)	21 (50.0)
Hemert	
Direct postop (*N*; %)	0 (0.0)
Vascular phase (*N*; %)	0 (0.0)
Osteoblastic phase (*N*; %)	8 (9.5)
Consolidation phase (*N*; %)	44 (52.4)
Cloudy bone formation (*N*; %)	28 (33.3)
BCP disappearing (*N*; %)	4 (4.8)
Varus angle (degrees; mean ± SD)	12.3 ± 2.3
Range of motion	
Flexion (degrees; mean ± SD)	120.8 ± 12.0
Extension (degrees; mean ± SD)	1.2 ± 2.1
KSS	89.8 ± 2.0
KSS functional	92.7 ± 1.9
Charlson comorbidity index	1.1 ± 1.1
JLCA	4.7 ± 2.2
LDFA	88.9 ± 2.0
MPTA	81.3 ± 2.4

Abbreviations: BCP, biphasic calcium phosphate; BMI, body mass index; JLCA, joint line convergence angle; KSS, Knee Society Score; LDFA, lateral distal femoral angle; mpta, medial proximal tibial angle; SD, standard deviation.

### Clinical results

The Knee Society Score (KSS) and Knee Society Functional Score assessment (KSFS) are critical parameters for evaluating post‐operative knee functionality. Our quantitative analysis demonstrated mean values of 89.8 ± 2.0 and 92.7 ± 1.9 for KSS and KSFS, respectively. Within the cohort, the Charlson comorbidity index—a prognostic tool estimating 10‐year survival in patients with comorbid conditions—was determined to have a mean score of 1.1 ± 1.1. Furthermore, radiographic metrics assessing lower extremity alignment, including the joint line convergence angle (JLCA), lateral distal femoral angle (LDFA) and medial proximal tibial angle (MPTA), revealed post‐operative mean measurements of 4.7 ± 2.2, 88.9 ± 2.0 and 81.3 ± 2.4, respectively, as detailed in Table 1. Univariate analysis about demographics revealed that there was no statistically significant difference between the two groups about age (36.29 ± 4.63 vs. 37.00 ± 5.49, *p* = 0.45), gender (male/female 12/9 vs. 13/8, *p* = 0.96) and BMI (26.9 ± 2.3 vs. 27.9 ± 1.8, *p* = 0.11). Patient‐reported outcome measures, specifically the WOMAC and VAS scores, demonstrated significant improvements at 12 months post‐operatively in both groups (*p* < 0.05). The WOMAC questionnaire encompasses pain, stiffness and physical function. Statistically significant improvements were observed in post‐operative comparisons across all subdomains in both groups (*p* < 0.05), except for stiffness in the allogenic graft group (*p* = 0.087) (Table 2).

**Table 2:** Pre‐ and Post‐op comparison.

Variables	Pre‐op	Post‐op			
		TCP graft	*p*	Allograft	*p*
WOMAC	16.1 ± 8.4	9.3 ± 4.8	0.000	11.3 ± 4.7	0.000
Pain	4.4 ± 1.7	2.4 ± 1.4	0.000	2.9 ± 1.3	0.000
Stiffness	1.7 ± 1.2	1.3 ± 1.1	0.015	1.6 ± 1.2	0.087
Physical function	9.9 ± 7.1	6.4 ± 4.0	0.000	7.7 ± 3.9	0.000
VAS	2.8 ± 0.9	1.8 ± 1.2	0.000	2.2 ± 0.8	0.000

Abbreviations: TCP, tricalcium phosphate; VAS, visual analogue scale; WOMAC, Western Ontario and McMaster University Osteoarthritis Index.

Post‐operative WOMAC scores, including subdomains (except pain, *p* = 0.305) and VAS scores, were comparable between the two groups (*p* < 0.05) (Table 3).

**Table 3:** Post‐op comparison.

Variables	TCP graft	Allograft	*p*
WOMAC	9.3 ± 4.8	11.3 ± 4.7	0.032
Pain	2.4 ± 1.4	2.9 ± 1.3	0.305
Stiffness	1.3 ± 1.1	1.6 ± 1.2	0.005
Physical function	6.4 ± 4.0	7.7 ± 3.9	0.000
VAS	1.8 ± 1.2	2.2 ± 0.8	0.020

Abbreviations: TCP, tricalcium phosphate; VAS, visual analogue scale; WOMAC, Western Ontario and McMaster University Osteoarthritis Index.

### Radiological results

**Figure 1:**
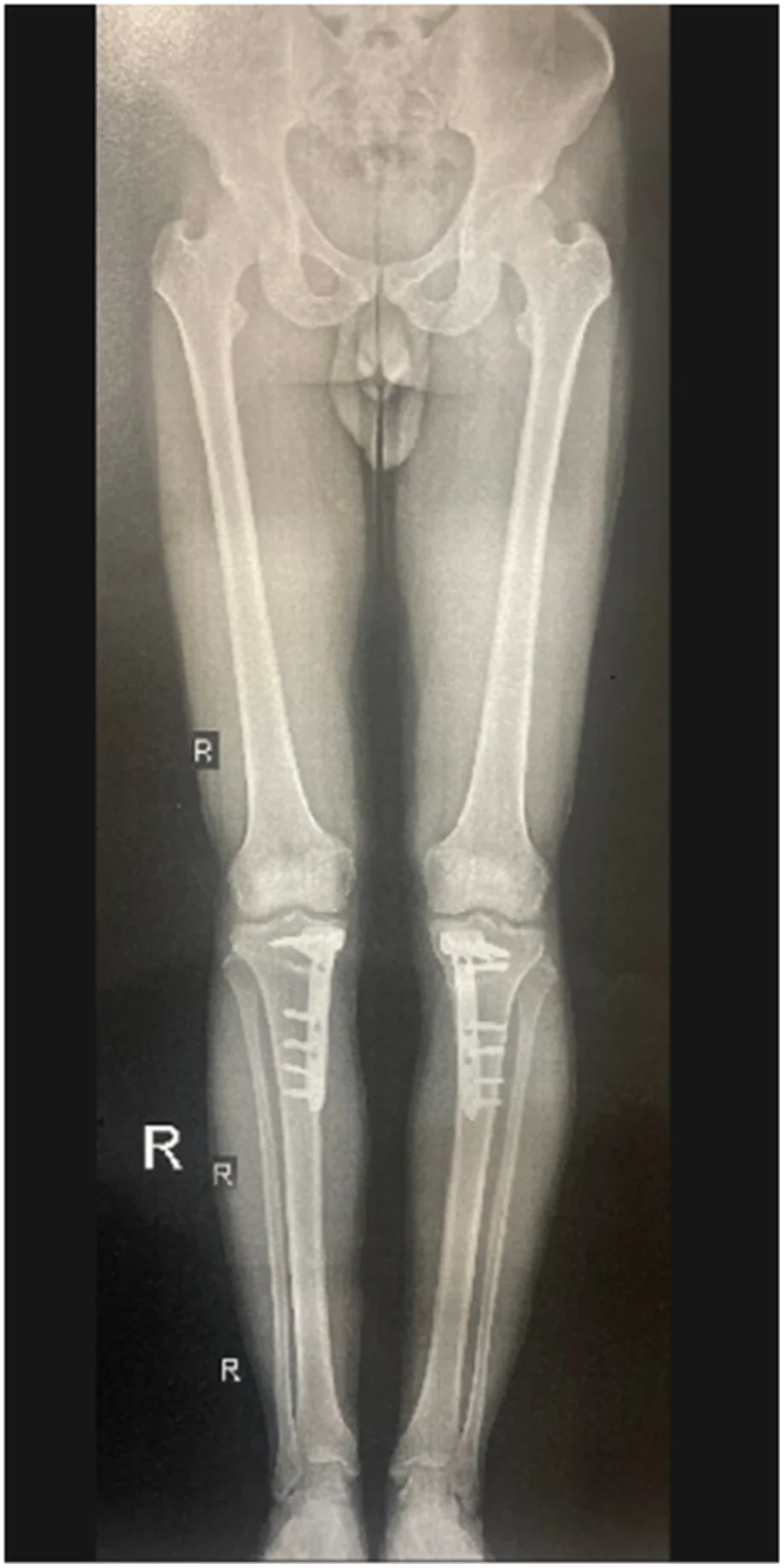
Post‐op x‐ray, left Knee 13 months after surgery with allogeneic bone graft, and right knee 10 months after surgery with TCP graft. TCP, tricalcium phosphate.

The pre‐operative mean varus angle was 12.3 ± 2.3° (range: 7–18°) (refer to Table 1). Post‐operative union assessment indicated that the majority of cases were in the consolidation phase of bone fusion (54.8%) (Figure 1), followed by cloudy bone formation (31%) (Table 1).

### Complications

There were no intraoperative complications. Throughout all stages of clinical follow‐up, there were no incidences of infection, other complications or radiographic evidence of loss of correction.

## DISCUSSION

The paramount finding of this investigation was the statistically significant disparities of pain and joint‐specific symptomatology between the utilization of TCP granules and allografts as osteoconductive fillers in OWHTO. TCP granules are a viable alternative to allogeneic bone grafts, which can elicit an immune response and pose a rare risk of disease transmission [2, 45]. When stabilized with rigid plate fixation, granule‐type bone grafts demonstrated no loss of correction at the 1‐year post‐operative mark, aligning with outcomes observed with wedge‐type bone grafts employed alongside locking plates [49]. The inherent brittleness of wedge blocks complicates their trimming and precise fitting into defects. Moreover, wedge blocks are prone to delayed resorption, potentially leading to delayed union and challenges in revision osteotomy or conversion to total knee arthroplasty [14, 48].

The trajectory of graft examination in studies is well‐defined; given the nature of this research and the significance of synthetic grafts, comparisons were made between these grafts and autografts, followed by allografts, and subsequently among various synthetic grafts (including different compositions such as hydroxyapatite, beta‐TCP and other forms like wedges, chips or granules) with autografts and allografts. Early studies indicated that the union rate was slower in patients where grafts were not utilized [4, 29].

Despite autologous cancellous bone grafts being regarded as the most efficacious graft material, their limited harvestable quantity has necessitated exploring and adopting alternatives [27]. Allografts, devoid of donor site morbidity associated with autografts, have shown comparable clinical and radiological outcomes in comparative studies [35]. Some investigations have reported satisfactory OWHTO outcomes without bone grafting [10, 43]. Nonetheless, allografts are prevalently employed as bone implants in OWHTO as substitutes for autografts [21, 41]. Concurrently, synthetic bone substitutes are under active research and development to mitigate risks associated with allografts, such as immune response or disease transmission. Reviews of other studies comparing synthetic grafts and allografts have shown promising results, with studies like that of Lee et al. [19] demonstrating similar clinical and radiological outcomes post‐OWHTO for both graft types. Other studies corroborated these findings, revealing that bone union with highly porous beta‐TCP granules increased linearly as post‐operative time progressed and was statistically significant compared to cancellous allogeneic bone chips [21, 34].

A comprehensive review of previous investigations examining parameters associated with knee functionality and activity delineates that the findings of the present study are concordant with those reported in other scholarly works. For instance, Montilla et al. [28] documented post‐operative outcomes of 93.5 ± 10.3, while Franceschetti et al. [11] observed values of 85.6 ± 14.7, underscoring the consistency and comparability of these findings with the current research. Furthermore, metrics pertinent to knee alignment, such as the JLCA, MPTA and LDFA, evidently indicate that subjects in this study attained clinically favourable outcomes. In this regard, the present investigation parallels the outcomes elucidated in analogous studies [15, 25, 44].

TCP is a synthetic bone substitute frequently employed as a grafting material in OWHTO [3]. Numerous studies have corroborated the efficacy of TCP as a bone graft substitute in OWHTO [24, 34, 36]. TCP has demonstrated biocompatibility, resorption and osteoconductive properties in a study utilizing β‐TCP wedge blocks in 16 patients undergoing OWHTO [12]. Histological and bone union assessments via bone biopsy post‐OWHTO indicated the effectiveness of TCP as a bone substitute in this study and some other studies [32, 40]. However, these studies were a case series with a limited sample size, no control group and lacked clinical evaluation. Due to the invasiveness, difficulty and potential complications associated with biopsy, subsequent studies employed radiographic alternatives to evaluate union status and surgical complications.

Some prior studies compared two synthetic bone substitutes, evaluating the bone union rates and absorbability of TCP and hydroxyapatite spacers. The findings revealed superior osteoconductivity and absorption rates in the TCP group compared to the hydroxyapatite group. The authors concluded that the excellent absorption of TCP facilitated future revision surgeries. However, the study's retrospective design and small patient cohorts in each group were limitations [7, 9, 29, 31]. This investigation, structured as a prospective randomized controlled trial, encompasses a statistically robust sample size, determined through comprehensive power analysis to ensure adequate reliability of outcomes. The sufficiency of our cohort is further substantiated by comparative reference to prior peer‐reviewed studies with smaller sample populations, including those conducted by van Hemert et al. [46] (*n* = 27), Onodera et al. [31] (*n* = 38), Jeon et al. [14] (*n* = 27), Putnis et al. [33] (*n* = 15) and Lee et al. [21] (*n* = 33). These precedents underscore the relative methodological strength and external validity of our study design. More studies need to be conducted to examine patient activity status post‐OWHTO, which we assessed using the WOMAC score. Various graft types have been evaluated in a few studies concerning patient performance. In fewer studies, grafts were compared within the same patient, with typically different graft populations being studied. One advantage of our research was the intra‐patient comparison of two graft types: an allograft used on one lower limb side and a synthetic graft on the other. This method mitigates bias related to pain, union process, health condition and performance status.

According to our investigations, only two studies have compared allogeneic and synthetic grafts using the WOMAC questionnaire to assess patient performance status. Despite the superior results of synthetic grafts over allografts, both studies indicated no statistically significant differences between the two groups [19, 20]. The superior results of synthetic grafts in our study suggest gradual improvement and bolster optimism for the future use of synthetic grafts.

This study has several limitations. There was no negative control group (receiving no graft). The follow‐up period was relatively short, with only four knees (4.8%) achieving grade six union, representing complete remodelling of the osteotomy site, at the 1‐year follow‐up. However, no differences in clinical or radiological outcomes were observed at each visit during the year, and no complications were detected. Despite these limitations, this study suggests that TCP granules can be a viable bone substitute, addressing some disadvantages of allogeneic bone grafts.

## CONCLUSION

Patients undergoing OWHTO utilizing TCP granules exhibited superior clinical outcomes, reduced post‐operative pain and diminished joint stiffness. These improvements were more pronounced in comparison to those treated with allogeneic bone grafts. Furthermore, the incidence of complications, post‐operative loss of correction and physical function remained comparable between the two cohorts at the 1‐year follow‐up, underscoring the efficacy and safety of TCP as a grafting material in the surgical management of genu varum deformity.

## AUTHOR CONTRIBUTIONS

**Salar Baghbani**: Conceptualization. **Yoosef Mehrabi**: Data curation. **Seyyed Reza Sharifzadeh**: Investigation. **Saeed Hesaraki**: Methodology. **Saeed Bakhshi**: Formal analysis; writing—original draft. **Davood Feizi**: Project administration. **Mostafa Shahrezaee**: Supervision. **Mohammad Movahedinia**: Writing—review and editing.

## CONFLICT OF INTEREST STATEMENT

The authors declare no conflicts of interest.

## ETHICS STATEMENT

Institutional Review Board approval was secured from our affiliated tertiary care academic hospital (Besat Hospital Review Board), and the approval number was IR.AJAUMS.REC.1402.127.Patients who were candidates for corrective proximal tibial osteotomy surgery using the OWO method according to the inclusion criteria were included in the study and underwent surgery after obtaining written informed consent.

## Data Availability

Data available on request from the authors.
